# The Silkworm as a Source of Natural Antimicrobial Preparations: Efficacy on Various Bacterial Strains

**DOI:** 10.3390/antibiotics10111339

**Published:** 2021-11-02

**Authors:** Maristella Mastore, Silvia Quadroni, Sara Caramella, Maurizio Francesco Brivio

**Affiliations:** 1 Laboratory of Comparative Immunology and Parasitology, Department of Theoretical and Applied Sciences, University of Insubria, 21100 Varese, Italy; maristella.mastore@uninsubria.it (M.M.); sara.caramella97@gmail.com (S.C.); 2 Laboratory of Ecology, Department of Theoretical and Applied Sciences, University of Insubria, 21100 Varese, Italy; silvia.quadroni@uninsubria.it

**Keywords:** antimicrobial peptides, *Bombyx mori*, natural compounds, silkworm, thermal stability, hemolymph

## Abstract

The global spread of multi-resistant pathogens responsible for infections, which cannot be treated with existing drugs such as antibiotics, is of particular concern. Antibiotics are becoming increasingly ineffective and drug resistance is leading to more difficult-to-treat infections; therefore, new bioactive compounds with antimicrobial activity are needed and new alternative sources should be found. Antimicrobial peptides (AMPs) are synthesized by processes typical of the innate immune system and are present in almost all organisms. Insects are extremely resistant to bacterial infections as they can produce a wide range of AMPs, providing an effective first line of defense. The AMPs produced by insects therefore represent a possible source of natural antimicrobial molecules. In this paper, the possibility of using plasma preparations from silkworm (*Bombyx mori*) larvae as a source of antimicrobials was evaluated. After simple purification steps, insect plasma was analyzed and tested on different Gram-positive and Gram-negative bacterial strains. The results obtained are encouraging as the assays on *Escherichia coli* and *Enterobacter cloacae* showed significant decrease in the growth of these Gram-negative bacteria. Similar results were obtained on Gram-positive bacteria, such as *Micrococcus luteus* and *Bacillus subtilis*, which showed strong susceptibility to the silkworm AMPs pool. In contrast, *Staphylococcus aureus* displayed high resistance to *Bombyx mori* plasma. Finally, the tested plasma formulations were assessed for possible storage not only at 4 °C, but also above room temperature. In conclusion, partially purified plasma from silkworm could be a promising source of AMPs which could be used in formulations for topical applications, without additional and expensive purification steps.

## 1. Introduction

Drugs with antibiotic activity are essential in the treatment of infections; however, overuse of these drugs in recent decades has led to the emergence of treatment-resistant strains of bacteria [1]. Moreover, most of the known drugs were developed between the 1940s and 1970s, after which innovations in this field were limited to chemical modifications of pre-existing molecules [2].

There is therefore an urgent need to identify new molecules and alternative sources, and to produce and test compounds with antibacterial activity as alternatives to conventional antibiotics, without inducing resistance. In this regard, antimicrobial peptides (AMPs) could be a valuable support to conventional antibiotic therapies [3,4]. These innovative and promising molecules are natural compounds showing a specific biological antimicrobial activity. They have been isolated from many species of plants, insects and other organisms, and derive directly from the mechanisms of the innate immune response [5,6].

AMPs are amphipathic peptides that generally have a low molecular mass, and they are produced by organisms in response to the presence of potentially pathogenic agents [7].

Although some parasites and pathogenic microorganisms are able to evade and depress the innate immune defenses of arthropods [8,9,10], insects, in particular, normally react to infections by activating their effective innate immune system. The insect’s immune system can be divided into cellular defenses, mediated by immunocompetent cells, and humoral defenses involving all the molecules that, once synthesized, are released into the circulatory stream and participate both in the immune recognition and in the effector phase responsible for neutralizing the non-self [11].

Insects are one of the possible and promising sources of AMPs; since 1974, more than 150 AMPs have been extracted and identified in insects [12,13]. In general, insects produce a repertoire of AMPs which usually possess similar structural characteristics, but often they are specific to certain classes of microorganisms [14]. The concurrent presence of several AMPs acting synergistically in a single individual can provide insects with an enhanced defense against unicellular invaders such as bacteria, fungi and protozoa [15].

AMPs are considered inducible factors, present only after infection; their synthesis is mediated by circulating cells, such as some hemocytes, but mainly by fat body cells. However, AMPs may be constitutively present as a local defense, at the level of epidermis, trachea midgut, and tissues of the reproductive organs [16,17,18].

AMPs typically consist of 12–50 amino acids and are classified according to their composition and amino acid sequence, and in most cases the hydrophobic regions of these molecules extend beyond half of the amino acid residues. These peptides/proteins possess various secondary structures such as: α-helices; β-strands, β-hairpins or loops, or an extended conformation [13,19]. They can be grouped according to their structure; 6–8 cysteine residues with disulfide bridges and terminal loop domains characterize defensins, a structural linearity without cysteine residues is typical of cecropins, while other peptides reveal a high proline and/or glycine content [20].

As mentioned above, although differences in amino acid sequence exist in the various orders of insects, there is a high rate of conservation in the structure of AMPs [21]. Their physico-chemical properties facilitate the interaction of the phospholipid bilayer with the pathogen membrane through electrostatic interactions with cell surface charges [22,23]. AMPs damage microorganisms as a result of membrane alteration due to mechanisms that in some cases are not yet fully elucidated. However, not all AMPs interact with membranes; some have intracellular targets, such as polyanionic molecules like DNA and RNA, enzymes in biosynthetic pathways, or proteins involved in folding control [24].

Some authors have described more than 30 AMPs in silkworms, classified into groups such as cecropins, attacins, moricins, gloverins, lebocins, enbocins, and defensins; most of these AMPs are effective against both Gram-positive and Gram-negative bacteria, as well as other microorganisms [25,26,27].

Different combinations of AMPs induced after infection are present in other insects, which synergistically contribute to bacterial clearance processes [28,29,30].

In this work, the silkworm *Bombyx mori* was used as a model organism to test the suitability of the hemolymph as a source of AMPs; the antimicrobial activity was evaluated by a track-dilution test of hemolymph fractions against *Escherichia coli*, *Enterobacter cloacae*, *Micrococcus luteus*, *Bacillus subtilis* and *Staphylococcus aureus*. Moreover, a preliminary assay of the thermal stability of hemolymph samples was carried out to assess their possible storage in non-refrigerated conditions.

## 2. Results

### 2.1. Effects of the Whole Plasma from B. mori Larvae on E. coli

**Figure 1:**
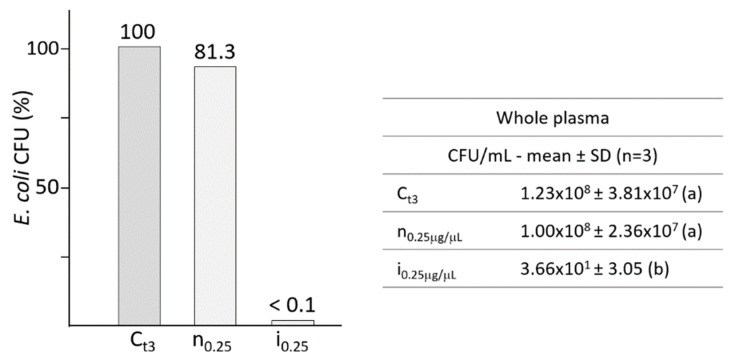
Bacterial growth of *E. coli* in the presence of the humoral fraction of whole hemolymph (0.25 μg/μL), from not infected (n) and infected (i) *B. mori* larvae. The bacterial growth after 3 h of incubation in culture broth was considered as the control (C_t3_), assuming it as 100% of survival. The table resumes the growth of *E. coli* expressed as CFU/mL. Different letters within brackets (a,b) indicate significant differences (*p* < 0.05 Tukey test). C: control; n: plasma from not infected larvae; i: plasma from infected larvae.

Silkworm hemolymph was collected from fifth larval instar and treated to prepare the plasma (cell-free) by low-speed centrifugation, then aliquots of the supernatants were incubated with *E. coli* and bacterial growth was assessed by plating and track-dilution assay (Figure 1).

Figure 1 shows the effects of 0.25 μg/μL of whole hemolymph on the growth of *E. coli*; samples from not infected larvae (n) induced a slight and not significant decrease in bacterial growth compared to the control, while incubation with samples obtained from infected larvae (i) led to almost complete mortality of *E. coli*.

**Figure 2:**
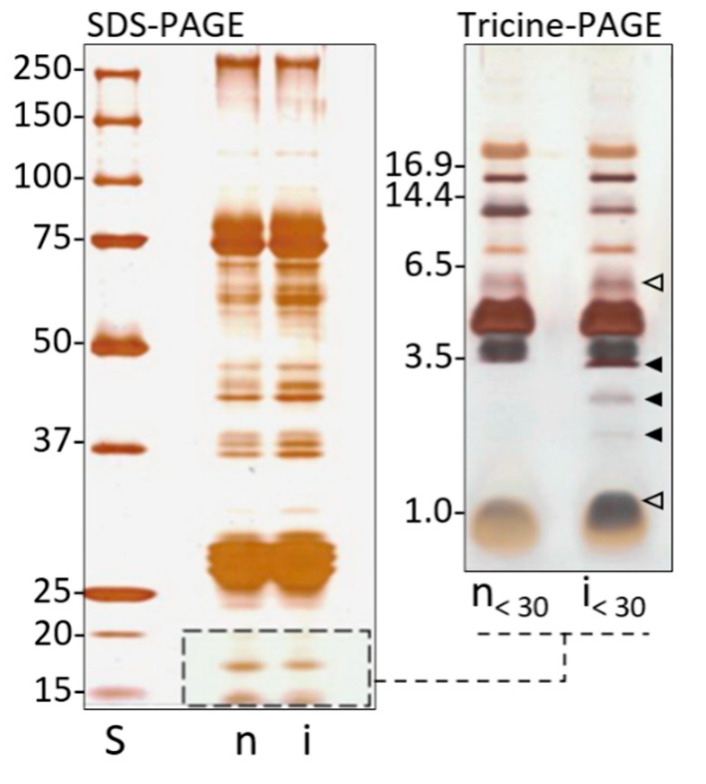
Analytical electrophoresis of hemolymph from not infected (n) and infected (i) *B. mori* larvae. On the left: SDS-PAGE (10%) of 30 μg/well (total proteins) of whole hemolymph. On the right, samples were analyzed by 16% Tricine-PAGE. Full arrowheads indicate low molecular weight inducible components newly synthesized after infection; empty arrowheads indicate quantitatively inducible low molecular weight components increased after infection. S: standard molecular weights.

Whole hemolymph from not infected and infected larvae was analyzed by SDS-PAGE (Figure 2, left); the protein pattern reveals a series of bands ranging from 270 to 10 kDa. Since the low molecular weight region (<20 KDa), typical of AMPs, is not clearly analyzable by this method, the samples were further separated by Tricine-PAGE (Figure 2, right). After hemolymph fractioning, samples < 30 kDa, from not infected (n < 30) and infected (i < 30) larvae were reanalyzed. A pattern of peptides from 20 to 1 kDa was observed; in particular, three bands (3.4, 2.8, 2.1 kDa) appeared to be synthesized ex novo; in addition, two bands (5.5 and 1.2 kDa) were quantitatively increased.

### 2.2. Effects of Fractioned Plasma from B. mori Larvae on Gram-Negative Bacteria

The silkworm plasma from not infected or infected larvae was previously centrifuged to isolate the <30 kDa fraction; various concentrations of low molecular weight protein pools (0.075, 0.15 and 0.25 μg/μL) were then assayed on *E. coli* and *E. cloacae*.

**Figure 3:**
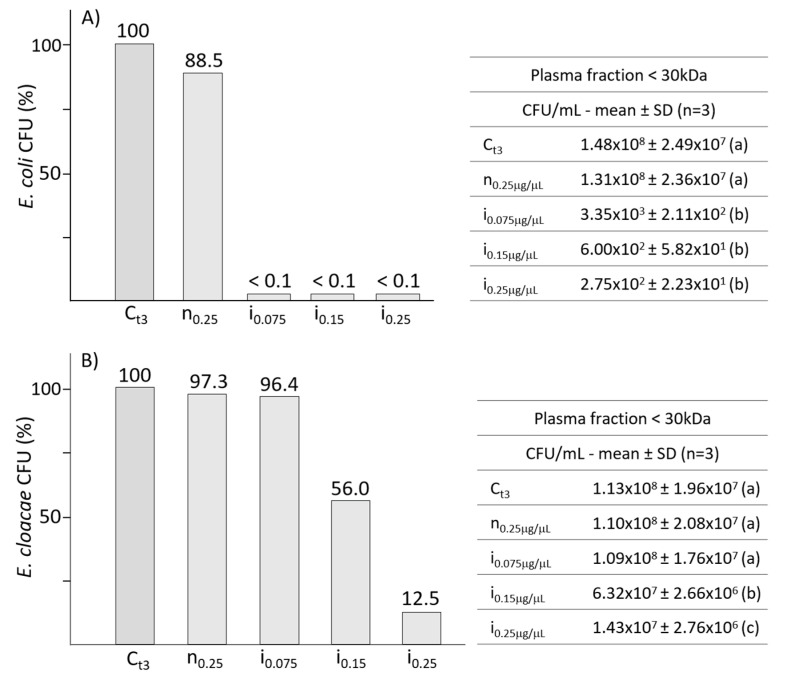
Panels (**A**,**B**) show the effects of various concentrations of silkworm AMPs on *E. coli* and *E. cloacae*, respectively. Tables on the right present the bacterial growths expressed as CFU/mL. Different letters within brackets (a,b,c) indicate significant differences (*p* < 0.05, Tukey test). C: control; n: plasma from not infected larvae; i: plasma from infected larvae.

The presence of AMPs from infected larvae at all the tested concentrations, showed similar bactericidal effects on the growth of *E. coli* (Figure 3, panel A), all leading to a significant decrease of CFU. Data from the graph in panel A show that the growth of *E. coli* was drastically inhibited even at the lowest concentration (0.075 μg/μL). The control from not infected larvae induced a slight and not significant reduction of bacterial growth.

When assayed on *E. cloacae,* the AMPs pool (Figure 3, panel B) showed a significant bacterial mortality at the concentrations of 0.15 and 0.25 μg/μL, reducing the growth to 56% and 12% respectively. The graph in panel B indicates that *E. cloacae* is less susceptible than *E. coli* to the action of AMPs; a marked decrease of *E. cloacae* growth was observed only at the concentration of 0.25 μg/μL.

### 2.3. Effects of Fractioned Plasma from B. mori Larvae on Gram-Positive Bacteria

**Figure 4:**
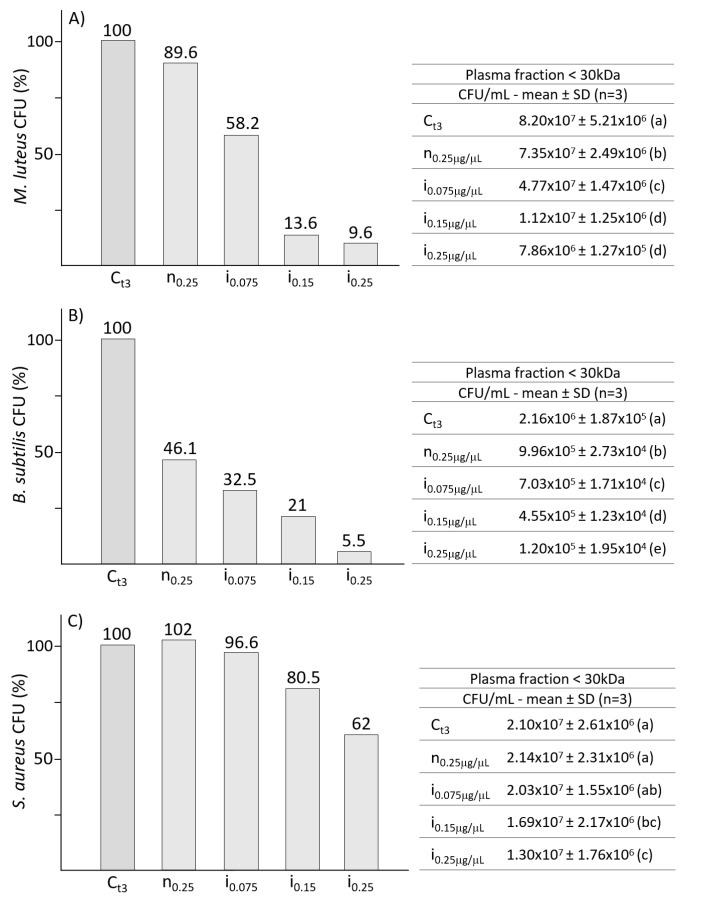
Panels (**A**–**C**) show the efficacy of the silkworm fractioned plasma on Gram-positive bacteria strains *M. luteus*, *B. subtilis* and *S. aureus*, respectively. Tables on the right present the bacterial growths expressed as CFU/mL. Different letters within brackets (a,b,c,d,e) indicate significant differences (*p* < 0.05, Tukey test). C: control; n: plasma from not infected larvae; i: plasma from infected larvae.

The efficacy of silkworm AMPs pool was tested on Gram-positive bacteria (Figure 4); fractioned plasma was incubated with *M. luteus* (panel A), *B. subtilis* (panel B) or *S. aureus* (panel C), and CFU count was performed by the track-dilution test.

A high efficiency in inhibiting the growth of *M. luteus* (Figure 4, panel A) was observed with concentrations of 0.15 μg/μL and 0.25 μg/μL, leading to a significant reduction of growth to 13.6% and 9.6% respectively.

An even greater efficacy was observed with the tests on *B. subtilis* (Figure 4, panel B), from which, already at the concentration of 0.075 μg/μL, a significant reduction in growth was observed. Antimicrobial activity on *B. subtilis* was correlated with increasing plasma concentration. In addition, the bacteria also appear to be susceptible to silkworm plasma from not infected larvae (n_0.25_); incubation with the low molecular weight fraction (0.25 μg/μL) induced a significant growth reduction of about 50%.

*S. aureus* (Figure 4, panel C) shows greater resistance to *B. mori* antimicrobial factors when compared to the other two Gram-positive bacterial strains; the incubation with the highest concentration (0.25 μg/μL) of the AMPs pool induced a reduction of growth lower than 40%.

### 2.4. Influence of Storage Temperature on the Efficacy of B. mori AMPs Pool

**Figure 5:**
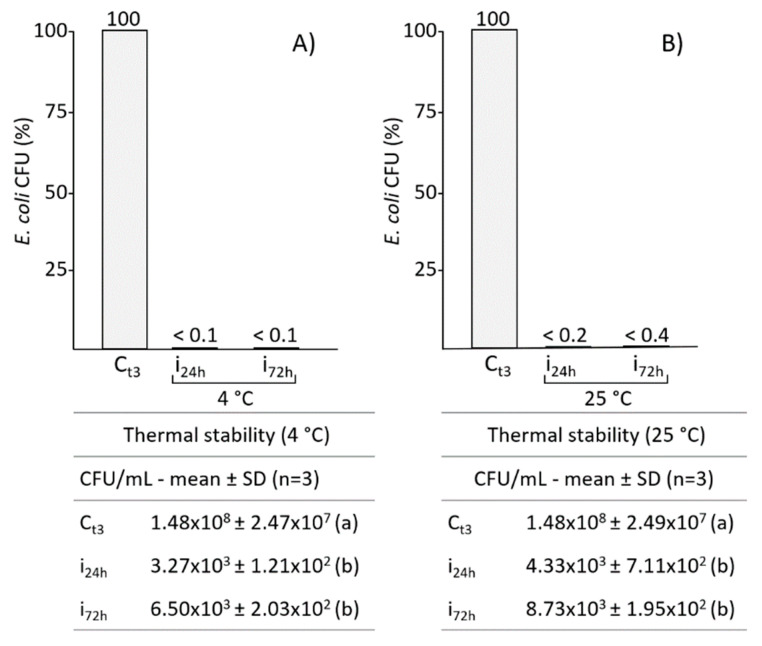
The activity of *B. mori* fractioned plasma, from infected larvae, was assayed for antibacterial properties after conservation at 4 (**A**) and 25 °C (**B**), for 24 and 72 h. Tables below present the *E. coli* growth expressed as CFU/mL. Different letters within brackets (a,b) indicate significant differences (*p* < 0.05, Tukey test). C: control; i: plasma from infected larvae.

In order to test whether temperature storage affects the effectiveness of the AMPs pool of *B. mori*, growth tests were carried out on *E. coli*, with samples previously stored for 24 and 72 h at both 4 °C and 25 °C. As expected, the data obtained show that storage at 4 °C did not affect the antimicrobial properties of the silkworm plasma, since a significant drastic reduction in the growth of *E. coli* was observed after both 24 and 72 h of storage at this temperature (Figure 5, panel A).

Also after 24 and 72 h storage at 25 °C (Figure 5, panel B), the efficacy of the AMPs pool remained very high.

## 3. Discussion

Several studies have been conducted on *B. mori*, ranging from the genetics of the insect, the immune response related to the health of the lepidopteran, and also its use as a source of high-quality proteins used in silk tissue manufacturing processes. This lepidopteran has also assumed great importance as a bioreactor capable of producing specific proteins such as silky biomaterials and molecules with biological activity [31]; the latter application is becoming particularly important in relation to the problem of the spread of different bacterial strains resistant to conventional antibiotics, and therefore, the lepidopteran is becoming important for the consequent search for natural compounds with antimicrobial activity [32]. Moreover, *B. mori* could be used as an alternative animal model for preclinical studies regarding infections by pathogens and the effectiveness of antibiotic therapies [32,33,34].

In this work, one of the main processes of the *B. mori* immune response that occurs after an infection was investigated; in particular, the presence and the efficacy of inducible factors such as AMPs on various bacterial strains were assessed. These molecules are closely related to infectious events and are synthesized via the specific intracellular pathways Toll and Imd [35]. The focus on AMPs comes from the proven antimicrobial activity of these peptides, since their properties make them potential candidates for alternative pharmacological approaches to antibiotic therapies [36,37].

Thus, the activity of a pool of antimicrobial molecules, present in whole and fractioned plasma collected from *B. mori* larvae, was assessed on different bacterial strains; the silkworm larvae were previously immunized with a mixture of Gram-positive and Gram-negative bacteria to ensure a broad-spectrum response by activating both Toll and Imd pathways.

**Figure 6:**
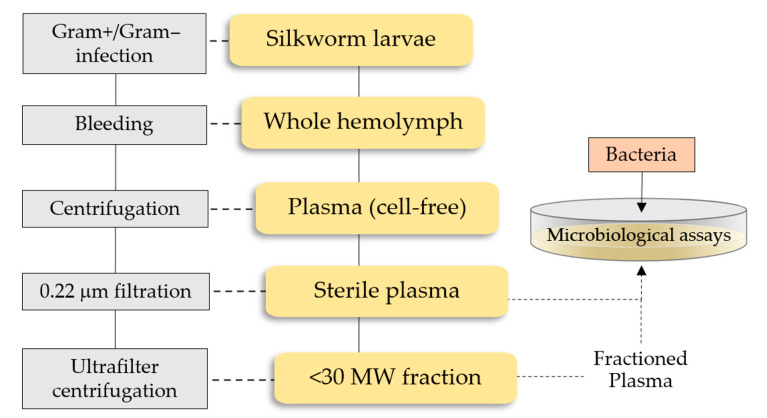
A schematic outline of the procedure to obtain the low molecular weight (MW) plasma fraction containing the silkworm AMPs pool.

In order to foresee possible future use in drug therapies, no further purification of insect plasma was conducted, with the aim to simplify procedures for the preparation of low-cost antibacterial formulations (Figure 6).

Specifically, to stimulate both AMPs synthesis pathways, larvae were co-infected with *E. coli* and *M. luteus*. After immunization, whole and fractioned plasma obtained from not infected and infected *B. mori* larvae were analyzed. A preliminary analysis of the whole plasma was performed by SDS-PAGE; although this method does not resolve the low molecular weight region in the protein pattern, some small proteins/peptides (below 20 kDa) were observed. Then, the fraction below 30 kDa was separated by Tricine-PAGE, which is specific for resolving low molecular weight compounds. In the plasma from infected larvae, the presence of several bands with a molecular mass lower than 6.5 kDa was observed, with some of them more represented than in the control from not infected larvae, and at least three bands appeared to be newly synthesized and at least two over synthesized.

Data from literature suggest that this peptide pool contains the main AMPs identified in *B. mori* [38], such as cecropins, defensins, lebocins, moricins, gloverins and attacins. Cecropins are cationic peptides that exhibit a broad range of antimicrobial properties against both Gram-positive and Gram-negative bacteria and fungi; defensins are cationic peptides rich in cysteine residues active against Gram-positive bacteria; lebocin is a proline-rich peptide acting in synergy with cecropin D, leading to the leakage of the lipid bilayer of bacterial membrane; moricin is a cationic peptide acting on various bacteria and fungi; gloverin is a glycine-rich peptide showing a weak activity against Gram-negative strains; and attacin exists in acidic and basic forms and appears to interfere with the synthesis of membrane proteins in Gram-negative bacteria, particularly *E. coli* [13,38,39]. The AMPs described above exhibit molecular masses that are consistent with the fraction < 30 kDa isolated and analyzed in *B. mori* in the present study.

Results from microbiological assays carried out with silkworm plasma have shown variable antimicrobial efficacy depending on the treated bacterial strain; *E. coli* was the bacterial strain most susceptible to treatments, in which even the lowest concentration induced high mortality. The growth of *E. coli* was completely inhibited when incubated for 3 h with 0.075 µg/μL of plasma peptides. Conversely, the Gram-negative *E. cloacae* showed a higher resistance to the AMPs of *B. mori*. The lower concentrations (0.075 and 0.15 µg/µL) did not induce relevant effects on the culture; only by incubation with 0.25 µg/μL did the growth drastically decrease to 12.5%. These data agree with the results by Wang et al. [40], who have shown that gloverin exerts antibacterial effects mainly on Gram-negative bacteria and fungi, with varying efficacy depending on the insect species considered. Moreover, since different microorganisms induce different immune signaling pathways, gloverins are generally considered to be induced through Toll and Imd pathways during immune responses. In contrast, only a few gloverins are known to exhibit inhibitory activity against Gram-positive bacteria. Furthermore, gloverin seems to have a synergistic activity when combined with cecropin A, suggesting that these two antibacterial peptides might employ different antimicrobial strategies and cooperate against microorganisms. Besides cecropin A, previous studies have also identified three other main cecropins (Cec B, D and E) in *B. mori*, showing a broad-spectrum action on both Gram-negative and Gram-positive bacteria [41]. The data obtained using the pool of *B. mori* AMPs also agree with the observations by Rahnamaeian et al. [42], who have suggested an enhanced action of multiple peptide combinations from hymenopterans, and their possible use as therapeutic agents against Gram-negative pathogens that have acquired resistance to common antibiotics. Accordingly, combinations of AMPs (defensins, cecropins, and diptericins) isolated from dipterans, such as *Calliphora vicina*, revealed significant efficacy against clinical strains of *E. coli*, *Klebsiella pneumonia*, and *Acinetobacter baumannii*; assays by Chernysh et al. [43] showed that preparations containing complexes of AMPs are more effective than single peptides, and also reduce the occurrence of bacterial resistance.

Regarding the treatments on Gram-positive strains, positive results were obtained on both *M. luteus* and *B. subtilis*. In particular, the former decreased its growth to 13.6 and 9.6% with 0.15 and 0.25 µg/μL of AMPs respectively; *B. subtilis* growth was inhibited in a stepwise manner that appeared to be correlated with the increase in AMPs concentration and showed a level of inhibition even when treated with plasma from not infected larvae, probably due to the presence of constitutive lysozyme in the hemolymph [44,45]. The Gram-positive strain having the greatest resistance to silkworm AMPs was *S. aureus*; even using the highest plasma concentration, *S. aureus* growth was never lower than 62%. The data on Gram-positive bacteria agree with the results by Hara and Yamakawa [46]; the presence of moricin in plasma, synthesized after bacterial infection, induces antibacterial activity against different bacteria strains and, in particular, seems to have greater activity against Gram-positive bacteria. Although cecropins have higher activity against Gram-negative bacteria, moricin and cecropins, simultaneously induced after a bacterial infection, can effectively eliminate a wide variety of invasive bacterial species.

As mentioned above, the efficacy of *B. mori* plasma hemolymph is higher on *B. subtilis* and *M. luteus* than on *S. aureus*. Accordingly, a low susceptibility of *S. aureus* to *Tenebrio molitor* AMPs was detected by Chae et al. [47], who have also suggested that tests performed with single purified peptides do not correctly reflect what happens at the physiological level, where bacterial clearance results from the action of multiple antimicrobial factors present in the hemolymph [48]. Even combinations of AMPs (CecA and cobatoxin) from *Galleria mellonella* (Lepidoptera) showed additive effects on *M. luteus* [49]; thus, such as in *B. mori*, also in the waxworm, combinations of antimicrobial compounds maximize the processes of microorganism elimination.

Other AMPs are present in *B. mori*, such as lebocins, defensins and attacins, that are generally effective against bacteria and fungi [50,51,52]. In particular, as suggested by Liu et al. [52], lebocin reveals a synergistic effect with Cec D; therefore, the presence of lebocin, which is able to alter the lipid bilayer of microorganisms, can support and amplify the action of cecropin.

From the obtained results, it is possible to assume that the antimicrobial effects observed using plasma from infected larvae may be attributable to the presence of both synthesized ex novo and over-synthesized proteins.

In this work, as well as in previous studies [47,48,49], the possibility that functionally distinct insect AMPs may act in a synergistic and/or additive manner, when expressed simultaneously following an infection, was highlighted.

The results obtained even with only a partial and inexpensive purification from *B. mori* hemolymph plasma showed promising antimicrobial activity, which could prelude to the preparation of low-cost formulations of pharmaceuticals for non-systemic use. It is known that AMPs cannot be administered parenterally, as they are neutralized by the patient’s immune response, but in dentistry, ophthalmology or dermatology applications, they could represent a valid alternative to the use of conventional antibiotics also against biofilm-producing bacteria [53].

In view of their possible use as topical drugs, the stability of the preparation at room temperature must be considered [54,55]. In this context, preliminary tests on the possible storage of these molecules under non-refrigerated conditions were carried out; the results obtained by keeping the preparations at temperatures above room temperature (25 °C) showed no significant loss of efficacy on *E. coli*.

In summary, the work presented, while anticipating further testing of antibiotic-resistant bacterial strains of nosocomial interest, provides a preliminary but promising perspective for the development of topically administered antimicrobial drugs.

## 4. Materials and Methods

### 4.1. Reagents and Instruments

All reagents were supplied by Sigma Chemicals (St. Louis, MO, USA), ICN (ICN Biomedicals, GmbH, Costa Mesa, CA, USA), and Merck Millipore Ltd. (Tullagreen, Cork, Ireland). Instruments were provided by Bio-Rad Laboratories (Detroit, MI, USA) and Celbio Spa (Milan, Italy, EU). Centrifugations were performed by a SIGMA 1-14 (SciQuip Ltd., Newtown, Wem, Shropshire, UK) microcentrifuge and by an Eppendorf 5804R (Eppendorf, AG, Hamburg, Germany) centrifuge. Spectrophotometric measurements were carried out using a Jasco V-560 (Jasco, Easton, MD, USA) spectrophotometer. All materials and buffers were autoclaved or filtered by 0.22 µm Minisart filters (Sartorius, Goettingen, Germany).

### 4.2. Insects Rearing

*B. mori* larvae were reared on mulberry leaves, in a climatic chamber at 25 ± 0.5 °C under a 12:12 h light: dark period and 70% relative humidity. Plasma was collected from chilling anesthetized larvae at the fifth larval instar (7th day age).

### 4.3. Bacterial Strains Culture Conditions

Gram-negative (*E. coli* C1a, *E. cloacae* ATCC 13047) and Gram-positive (*B. subtilis* ATCC 6051, *M. luteus* ATCC 4698, *S. aureus* ATCC 6538) bacteria were used for larval immunizations and AMP activity assays. After inoculation in Luria-Bertani (LB) broth (1% tryptone, 0.5% yeast extract, 0.5% NaCl), bacterial cultures were grown for 24 h under shaking (180 rpm) in dark condition, at an optimal growth temperature for each strain (37 °C for *E. coli*, *B. subtilis* and *S. aureus*; 28 °C for *M. luteus*; 30 °C for *E. cloacae*). Bacterial growth was evaluated by spectrophotometric measurement of biomass (λ = 600 nm). Briefly, immunization assays were carried out by a mixture of *E. coli* and *M. luteus* 1:1 (*v/v*); cultures were centrifuged at 1700× *g* for 10 min at 20 °C and the bacterial pellet was recovered, then cells were killed by heating in a thermostatic bath at 65 °C for 1 h. Before injections, bacteria were washed several times with sterile PBS (138 mM NaCl, 2.7 mM KCl, 10 mM Na_2_HPO_4_/KH_2_PO_4_, pH 7.4) and finally the bacterial mix was injected into *B. mori* larvae at a final concentration of 10^3^ CFU/larva, or each strain was stored at −20 °C with 20% (*v/v*) glycerol for further cultures.

### 4.4. B. mori Immunization and Plasma Collection

To induce the synthesis of AMPs, *B. mori* larvae were anesthetized on ice and infected with 10 µL containing 10^3^ CFU of the *E. coli*/*M. luteus* suspension in sterile PBS. After infection, larvae were maintained in a climatic chamber for 24 h in the dark, at 25 °C. All experiments were performed under sterile conditions and not infected larvae were used as controls.

Hemolymph plasma was collected from the fifth instar larvae. Larvae were surface sterilized with 70% ethanol and then washed in sterile PBS; anesthetized larvae were bled by puncture of a proleg, and hemolymph flushed out in a refrigerated sterile tube containing a few 1-phenyl-2-thiourea crystals to avoid undesired activation of the prophenoloxidase enzyme. Cell-free plasma was obtained by clarification and increasing centrifugations (up to 1500× *g*) to remove cells and tissue debris. Whole plasma was filtered on 0.22 µm Minisart or processed by Amicon^®^ Ultrafilters (Millipore, Burlington, MA, USA) to obtain low molecular mass fractions (cut-off 30 kDa). Total protein content was determined by Bradford protein assays calibrated on BSA. All samples were used immediately or stored at −20 °C.

### 4.5. Antimicrobial Activity in Whole and Fractioned B. mori Plasma Samples

Both whole and fractioned plasma samples were assayed for antimicrobial activity by track dilution assays [56]. Gram-negative (*E. coli* and *E. cloacae*) and Gram-positive (*B. subtilis*, *M. luteus* and *S. aureus*) bacteria cultures were diluted to a final concentration of 10^6^ CFU/mL with LB broth. For each treatment, 20 μL of whole plasma, or fractioned plasma, were added to 180 μL of bacteria culture. To evaluate the expected bacterial growth, 20 μL of PBS were added to the bacteria culture (180 μL). All samples were incubated for 3 h under shaking (180 rpm) at the optimal growth temperature of the tested bacteria. After incubation, 100 µL of each sample was placed in a well of a 96-MicroWell™ plate and samples were serially diluted with phosphate buffer (61.4 mM K_2_HPO_4_, 38.4 mM KH_2_PO_4_). Each dilution was plated on solid agar and incubated for 24 h more. Finally, bacteria colonies were counted. The antibacterial activity of hemolymph samples was expressed as percentage of bacterial survival compared to the control (bacterial suspension incubated for 3 h without *B. mori* plasma). The concentrations of total proteins, from not infected or infected plasma, used in the antimicrobial activity assays, were 0.075, 0.15 and 0.25 µg/µL. For each analysis and treatment, hemolymph of five larvae was extracted and the experiment was repeated three times.

### 4.6. SDS and Tricine-PAGE Analysis of Larvae Plasma

Whole and fractioned plasma (<30 kDa) were analyzed by mono-dimensional electrophoretic separation, 10% SDS-PAGE [57] and by 16% Tricine-PAGE [58], respectively.

Plasma from not infected and infected larvae were obtained as described in paragraph 4.4; before electrophoretic separation, samples were dialyzed against 10mM Tris-HCl, pH 7.2, overnight at 4 °C, and finally protein pools were precipitated with trichloroacetic acid (20% *v/v*). Samples were resuspended in SDS sample buffer or Tricine sample buffer, and denatured for 10 min at 100 °C. For each analysis, 30 µg/well was loaded on slab gels. Electrophoresis was carried out by a vertical Protean^®^ II xi Cell (Bio-Rad) at 50 V (constant voltage) overnight. After separation, protein patterns were detected by silver staining.

### 4.7. Assays of Thermostability of B. mori Fractioned Plasma

To assess the stability of plasma AMPs, samples < 30 kDa were stored at different temperatures (4 and 25 °C), for 24 and 72 h. After storage periods, the fractions were tested on *E. coli* by track dilution assay, to assess any changes in antimicrobial activity.

### 4.8. Data Processing and Statistical Analysis

Means and standard deviations (SD) were calculated in all assays. All experiments were replicated three times. For each assay, one-way analysis of variance (ANOVA), followed by Tukey test for pairwise comparison, was carried out to test for significant (*p* < 0.05) differences among controls and treatments. Data were processed with GraphPad Prism 8 (GraphPad Software, La Jolla, CA, USA).

## Data Availability

The data presented in this study are available upon request to the corresponding author.
